# Hepatocyte-secreted extracellular vesicles modify blood metabolome and endothelial function by an arginase-dependent mechanism

**DOI:** 10.1038/srep42798

**Published:** 2017-02-17

**Authors:** Felix Royo, Laura Moreno, Justyna Mleczko, Laura Palomo, Esperanza Gonzalez, Diana Cabrera, Angel Cogolludo, Francisco Perez Vizcaino, Sebastiaan van-Liempd, Juan M. Falcon-Perez

**Affiliations:** 1 Exosomes Laboratory. CIC bioGUNE, CIBEREHD, Derio 48160 Bizkaia, Spain; 2 Department of Pharmacology, School of Medicine, University Complutense of Madrid, CIBERES. Instituto de Investigación Sanitaria Gregorio Marañón (IISGM), 28040 Madrid, Spain; 3 Metabolomics platform, CIC bioGUNE, CIBERehd, Derio 48160 Bizkaia, Spain; 4 IKERBASKE Basque Foundation for science, Bilbao, 48013 Bizkaia, Spain

## Abstract

Hepatocytes release extracellular vesicles (EVs) loaded with signaling molecules and enzymes into the bloodstream. Although the importance of EVs in the intercellular communication is already recognized, the metabolic impact of the enzymes carried by these vesicles is still unclear. We evaluated the global effect of the enzymatic activities of EVs by performing untargeted metabolomic profiling of serum samples after their exposure to EVs. This approach revealed a significant change in the abundance of 94 serum metabolic signals. Our study shows that these vesicles modify the concentration of metabolites of different chemical nature including metabolites related to arginine metabolism, which regulates vascular function. To assess the functional relevance of this finding, we examined the levels of arginase-1 protein and its activity in the hepatic EVs carrying the exosomal markers CD81 and CD63. Remarkably, the arginase activity was also detected in EVs isolated from the serum *in vivo*, and this vesicular activity significantly increased under liver-damaging conditions. Finally, we demonstrated that EVs secreted by hepatocytes inhibited the acetylcholine-induced relaxation in isolated pulmonary arteries, *via* an arginase-dependent mechanism. In summary, our study demonstrates that the hepatocyte-released EVs are metabolically active, affecting a number of serum metabolites involved in oxidative stress metabolism and the endothelial function.

Hepatocytes, like most cell-types, secrete extracellular vesicles (EVs) into the extracellular *milieu*. Since their first description and characterization^1^, the interest in their possible biomedical applications has been growing exponentially. Most of the studies of EVs have used them to identify low-invasive disease biomarkers for diagnostic purposes (reviewed in ref. 2). However, the biological role of EVs is still mostly unknown. In the hepatic context, the first insights into their activity have been provided by the analysis of the EVs obtained from a liver-derived cell line with stem cell properties^3^. The EVs released by this cell line can induce the *in vitro* proliferation and apoptosis resistance of hepatocytes and stimulate the proliferation of tubular epithelial cells. *In vivo*, these EVs contribute to liver regeneration after experimental hepatectomy and acute liver damage^3^,^4^,^5^. Furthemore, it has been shown that hepatocyte-derived EVs deliver the enzyme sphingosine kinase 2 to form sphingosine-1-phosphate in the targeted hepatocytes, resulting in cell proliferation and liver regeneration after partial hepatectomy^6^. It has been recently reported that such EVs mediate the activation of macrophages during the development of non-alcoholic steatohepatitis^7^. They might also be involved in the initial development of drug-induced liver injury^8^,^9^. The available data support the notion that the hepatic EVs play an important role in different physiological and pathological processes.

Cells release different types of EVs, from 30 to 5,000 nm in size. The EVs comprise mainly the endosome-originated exosomes, plasma membrane-derived microvesicles, and apoptotic bodies, with different cargos and physiological properties^10^. Our group has demonstrated that the hepatocytes release small EVs (below 220 nm), which, in addition to known exosomal markers, contain over 200 various enzymes^1^,^11^ that cover a wide range of cellular pathways (Fig. 1). These enzymes are involved in many different processes, such as anabolism and catabolism of macromolecules and the energy and drug metabolism. This functional diversity makes it difficult to form an integrated view of the function of hepatocyte-derived EVs in the extracellular space.

Metabolomics based on ultra-high-performance chromatography coupled to mass spectrometry (UPLC-MS) is a sensitive technology for the simultaneous analysis of thousands of metabolites (i.e., molecules smaller than 2,000 Da)^12^. This technology offers the unique possibility to cover a wide spectrum of cellular and metabolic pathways by employing several metabolite extraction methods in combination with various chromatography conditions. Such comprehensive coverage allows the elucidation of mechanisms of action and functions of complex macromolecules in an integrated and unbiased manner^13^. We have recently reported by targeted metabolomics that hepatocytes-derived EVs are able to affect the serum levels of several lipids supporting the presence of active enzymes involved in lipid metabolism^14^. Here, we applied an untargeted metabolomics approach to study the global effect of the enzymatic activities carried by small EVs (secreted by primary rat hepatocytes) on the serum metabolic profile. Serum incubated with EVs showed significant changes in the abundance of several metabolites, including L-arginine, ornithine, citrulline, glutamate, 3-methyl-L-hisyidine, anserine, LPC(14:0), glycerophosphocholine, deoxyinosine, and ascorbic acid. The metabolic context of these molecules suggests the EV involvement in the metabolism of arginine, histidine, ammonia, lipids, purines, and in the oxidative metabolism. Interestingly, we demonstrated the presence of the enzyme arginase-1 (Arg1) in these vesicles, in accord with the reduced levels of arginine and increased levels of ornithine in samples incubated with EVs. This enzyme is responsible for the conversion of arginine into ornithine, a critical regulatory step in the synthesis of nitric oxide and the vascular function^15^. Finally, we also demonstrated that the small hepatic EVs regulated the endothelial function (assessed under acetylcholine stimulus) in dissected pulmonary arteries, *via* an arginase-dependent mechanism.

## Results

### Metabolic serum profiling

**Figure 2:**
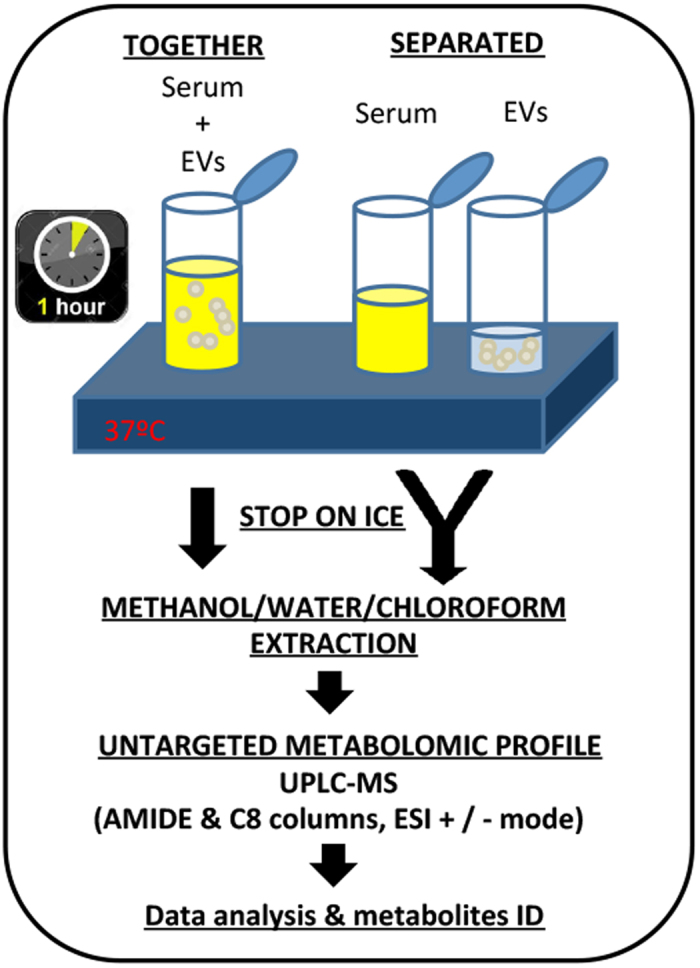
Untargeted metabolomics approach for studying the global metabolic effect of EVs secreted by primary rat hepatocytes. Rat serum samples were incubated for 1 h at 37 °C in the presence of small EVs secreted by primary rat hepatocytes (n = 7; each EV preparation was obtained from a different rat). As a control, in parallel, tubes containing rat serum or small EVs were separately incubated for 1 h at 37 °C, and combined after cooling down on ice (n = 7; in the case of serum each sample was an aliquot of pooled serum samples. Each EV preparation was obtained from a different rat). After incubation, polar and apolar metabolites were extracted using methanol/water/chloroform as indicated in the *Material and Methods*. Afterward, the extracted metabolites were analyzed using UPLC-MS, employing Amide and C8 chromatographic columns and positive and negative ESI mode. Data were processed to detect and identify the metabolites differentially expressed in the serum samples incubated at 37 °C together or separately with EVs.

**Figure 3:**
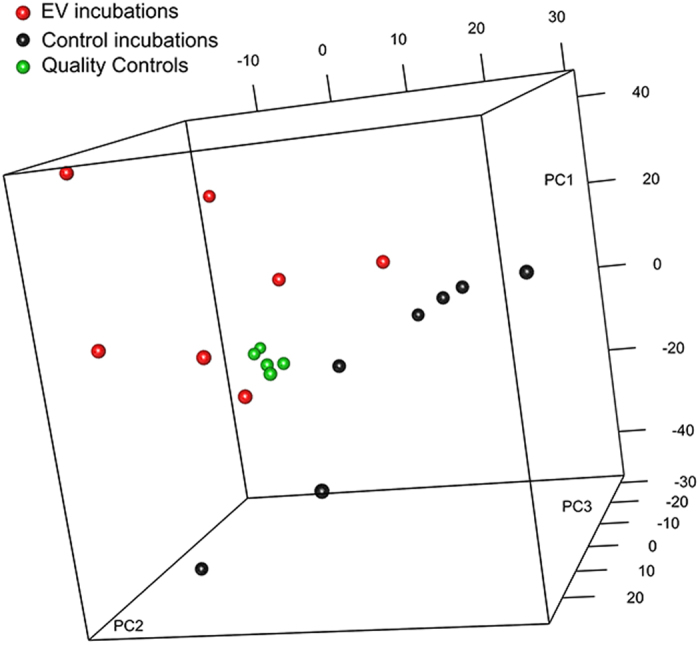
Metabolic effect of hepatocyte-secreted EVs. Principal component (PC) analysis of the serum metabolic profiles obtained by untargeted UPLC-MS metabolomic analysis of serum samples incubated with or without EVs (as described in the *Material and Methods*). Quality control (QC) samples are a pool of aliquots of all samples. QC samples are clustered together, indicating a good performance of the UPLC-MS system. *Note:* EVs induced sufficient changes in the serum metabolome to distinguish between the serum samples incubated at 37 °C together or separately with hepatic EVs.

To study the global enzymatic activity of hepatic EVs, rat serum samples were incubated for 1 h at 37 °C in the presence of 70 μg/ml (in all the cases referred to EV protein content) of small EVs secreted by primary rat hepatocytes (Fig. 2). In parallel, separate tubes containing rat serum samples or EVs were also incubated and used to exclude the metabolic changes not caused by the EVs. To achieve a robust statistical analysis, we used seven biological replicates with EVs obtained from the primary hepatocytes of seven independent rats. After the incubation, polar and apolar metabolites were extracted and analyzed using UPLC-MS, employing the Amide and C8 chromatographic columns as described in *Materials and Methods* section. This comprehensive UPLC-MS analysis allowed us to cover a wide range of molecules of the serum metabolome. We detected 5,732 metabolic signals; they were normalized by total peak area and corrected for QC time, depending on the intensity shift of the UPLC-MS system. Afterward, the data were visually inspected using the PCA, an unsupervised pattern recognition method initially used to discern the differences between spectral profiles (Fig. 3). The QC samples were clustered together, indicating a good performance of the UPLC-MS system. Interestingly, the PCA analysis also revealed that the metabolic profile discriminated serum samples that were incubated at 37 °C in the presence of EVs from the samples incubated without EVs. This result suggested that small EVs released from hepatocytes were metabolically active and affected the serum metabolome. Ninety-four metabolic peaks were differentially expressed in the two groups. Among them, we identified 12 serum metabolites whose levels were significantly modified by the incubation with EVs (Table 1). While the serum levels of some of these metabolites such as ascorbic acid, citrulline, arginine, and LPC(14:0) were significantly reduced, the levels of others (3-methyl-histidine, glutamyl-alanine, deoxyinosine, glutamate, glycerophosphocholine, anserine, ornithine, and methyl-arginine) were significantly enhanced by the enzymatic activity of the hepatic EVs (Table 1). The results of mapping these metabolites using the KEGG pathway database (Table 2) suggested an extracellular physiological role of the small hepatic EVs in the oxidative response, purine and protein synthesis, and in lipid, histidine, and arginine metabolism.

### Arginase-1 activity is associated with the hepatocyte-derived small EVs

**Figure 4:**
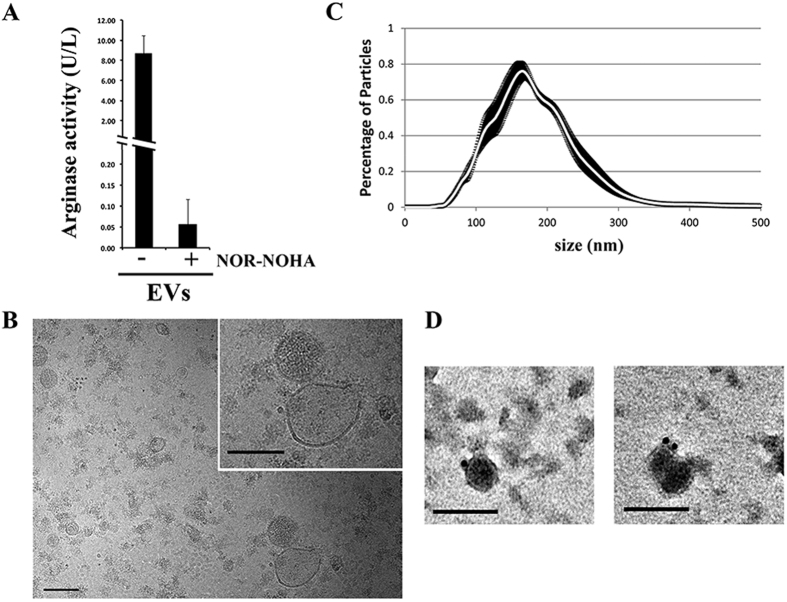
Arginase activity in small EVs secreted by primary rat hepatocytes. **(A)** Arginase activity was evaluated, using a colorimetric assay, in the absence or presence of arginase inhibitor NOR-NOHA (n = 2; *p* = 0.02; error bars represent S.D.). (**B–D**) EV preparations (n = 3) characterized using cryo-EM (**B**), nanoparticle-tracking analysis (**C**) and immuno-EM with an antibody against Arg1 protein (**D**) *Bar* = 100 nm.

**Figure 5:**
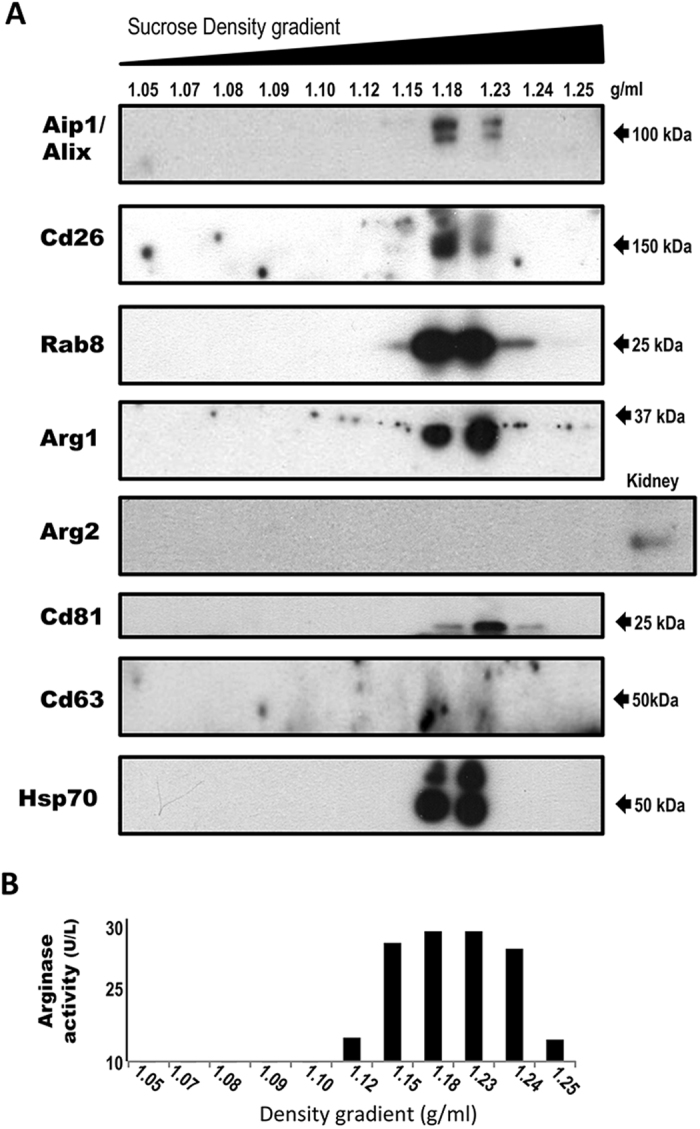
Arginase 1 is associated with exosomes-like vesicles. EVs secreted by primary rat hepatocytes were fractionated using a continuous sucrose density gradient. The fractions were assayed using Western blotting **(A)** with antibodies against indicated proteins and arginase activity **(B)**. *Note:* Arginase-1 protein and activity co-fractioned with exosomal markers Aip1/Alix, CD26, Rab8, CD81, CD63, and Hsp70.

Three (arginine, ornithine, and citrulline) out of the twelve serum metabolites whose levels were altered by the hepatic EVs belong to the arginine metabolism, a key regulator of nitric oxide levels and vascular function. The relative changes in the levels of arginine and ornithine accounted for 44% and 68% of the initial concentrations, indicating a strong effect of the hepatic EVs on the extracellular metabolism of these two amino acids. The transformation of arginine into ornithine is a well-known reaction catalyzed by arginases. The enzymes come from two sources. Arg1 is found in the cytosol of the hepatocytes, where it is involved in the urea cycle, a chain of reactions removing the toxic ammonium. Arginase 2 (Arg2) is located mainly in the mitochondria of extrahepatic tissues^16^. Our previous proteomics analysis study has reported the presence of Arg1 in small EVs derived from rat hepatocytes^1^. Here, our unbiased metabolomics approach showed that the hepatic EVs reduced the serum levels of arginine and increased the levels of ornithine (Table 1). To examine the arginase activity of EVs using an independent and easily accessible method, we employed a commercial colorimetric assay based on the production of urea, a sub-product of the conversion of arginine into ornithine. We detected significant arginase activity associated with the EV preparations (Fig. 4A). Notably, the EV-associated arginase activity was sensitive to the treatment with N^ω^-hydroxy-L-arginine (NOR-NOHA), an inhibitor of arginase^17^, confirming the presence of this specific activity in the EVs preparations. Cryo-EM analysis and nanoparticle-tracking analysis (NTA) confirmed the presence of membrane vesicles of 70–300 nm in size (Fig. 4B,C), and immuno-EM using an antibody against Arg1 further confirmed the association of this enzyme with a population of EVs (Fig. 4D). It is also worthy to note that in addition to vesicles, electron microscopy also showed other structures that may be compatible with different source of contaminating material such as protein aggregates. To obtain further confirmation of the presence of Arg1 protein and its activity in small EVs and to exclude the possible association with protein aggregates, we fractionated the EV preparation in a continuous sucrose density gradient (Fig. 5). The Arg1 protein and arginase activity were clearly detected in the same fractions as the exosomal protein markers Aip1/Alix, CD81, CD63, Rab8, CD26, and Hsp70. We detected the Arg2 protein in the kidney protein extract but not in the hepatic EV preparations (Fig. 5), indicating that the enzyme responsible for the arginase activity in the hepatocyte-secreted EVs is Arg1.

### Liver-damaging conditions increase EV-associated arginase activity *in vitro* and *in vivo*

**Figure 6:**
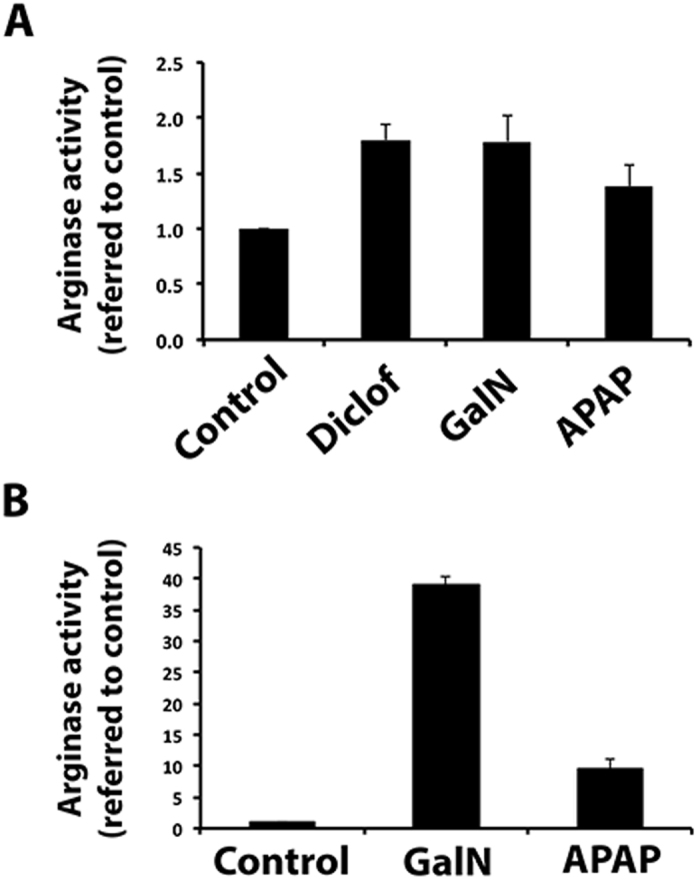
The increase in the level of vesicular arginase activity *in vitro* and *in vivo* under hepatotoxic conditions. **(A)** Arginase activity detected in EVs secreted by primary rat hepatocytes untreated (control) or treated with indicated drugs (n = 3; ANOVA *p* = 0.02; error bars represent S.E.M.). **(B)** Arginase activity detected in EVs isolated from serum of rats treated with vehicle (control) or indicated drugs (n = 3; ANOVA *p* < 0.001 error bars represent S.E.M.). In all cases, the arginase activity (U/L) was normalized to the activity values detected in the control.

We studied the hepatic EV-associated arginase activity under liver-damaging conditions. We examined the EVs released by untreated (control) primary rat hepatocytes and the hepatocytes treated with galactosamine, diclofenac, or acetaminophen (Fig. 6A). Arginase activity was significantly higher in EVs from hepatotoxicant-treated hepatocytes. Then, we checked whether this *in vitro* increase could also be observed *in vivo*, by extracting the serum and circulating EVs from the untreated and hepatotoxicant-treated rats. We observed a ten-fold increase in arginase activity in the serum of rats treated with different hepatotoxicants in comparison with the samples from untreated rats (Supplemental Figure 1A). The increase in the serum arginase activity was positively correlated with the serum glutamic-oxaloacetic transaminase (GOT) activity (Supplemental Figure 1B). A significant increase in arginase activity was also observed in circulating EVs isolated from the serum of rats treated with the hepatotoxicants (Fig. 6B).

Our results strongly support the idea that the hepatocytes secrete small EVs with exosomal features, containing active Arg1 enzyme. Importantly, under liver-damaging conditions, the hepatocyte-secreted EVs contain more arginase activity that could be systemically disseminated through the bloodstream, facilitating both paracrine and long-distance effects.

### Arginase inhibition prevents EV-induced pulmonary endothelial dysfunction

**Figure 7:**
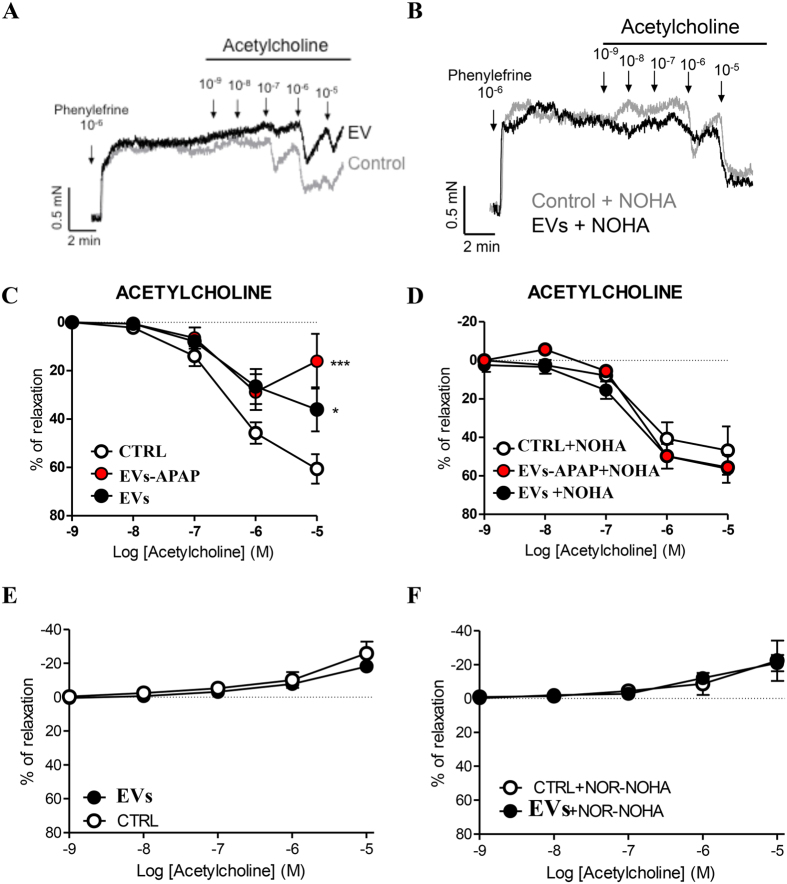
Pulmonary endothelial dysfunction induced by EVs is prevented by the inhibition of arginase. **(A**,**B)** Representative tracings showing the relaxant effects induced by acetylcholine (ACh) in the pulmonary arteries (PA). PAs were pre-incubated for 2 h under control conditions (control) or after treatment with EVs (50 μg/mL) in the absence **(A)** or presence **(B)** of the arginase inhibitor N^ω^-hydroxy-nor-L-Arginine (NOR-NOHA, 10 ng/mL) and mounted on a wire myograph. **(C**,**D)** Concentration-dependent relaxation induced by the endothelium-dependent vasodilator ACh in control (n = 7, different rats), APAP-EVs-treated (red dot, n = 4) or EV-treated (black dot, n = 6) rat PA rings incubated in the absence **(C)** or presence of NOR-NOHA **(D)**. **(E**,**F)** Concentration-dependent contraction induced by ACh following treatment with the eNOS inhibitor L-NAME in control or EV-treated rat PA rings incubated in the absence (E; n = 5) or presence of (F; n = 4) NOR-NOHA. Results are expressed as a percentage of the relaxation induced by ACh. *p < 0.05 versus control (repeated measures ANOVA followed by Bonferroni’s *post hoc* test). Error bars represent S.E.M.

**Figure 8:**
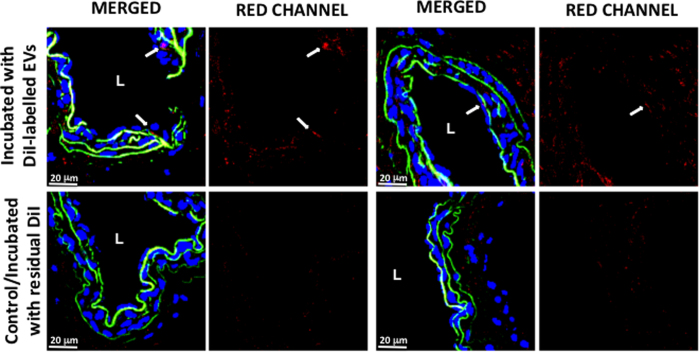
Incorporation of hepatic EVs into endothelium of pulmonary artery. Arteries were dissected and incubated with DiI-labeled EVs or residual non-vesicular DiI as described in *Materials and method* section. Two representative confocal images of each condition are depicted. DiI-label is in red, autofluorescence due to elastin is in green and DAPI (nucleus) is in blue. Lumen of the artery is indicated by “L” and white arrows pointed the presence of DiI-labeled EVs in the endothelium of the artery. *Bar*, 20 μm.

Finally, we evaluated the physiological effect of the vesicle-associated arginase activity. Arginine is the substrate for nitric oxide synthases. An increased arginase activity is associated with defective endothelial nitric oxide (NO) synthesis and activity, i.e., causes the endothelial dysfunction^18^. In agreement with our *in vitro* results, the endothelium-dependent relaxant responses to acetylcholine were significantly reduced in the pulmonary arteries (PA) exposed to hepatocyte-derived small EVs for 2 h (Fig. 7A,C). Remarkably, the EVs produced under liver damaging conditions (i.e. APAP treatment) showed higher endothelial dysfunction effects (Fig. 7C). These effects were fully suppressed by the arginase inhibitor NOR-NOHA (Fig. 7B,D). In contrast, none of the treatments affected the responses induced by ACh following the inhibition of eNOS by L-NG-nitroarginine methyl ester (L-NAME) (Fig. 7E,F) or the endothelium-independent relaxation induced by SNP (Supplemental Figure 2). Interestingly, under our experimental conditions we were able to detect EVs attached to the endothelium of the pulmonary artery. Since the confocal microscopy employing fluorescently labeled EVs only showed nucleus of the cells, we could not assure whether EVs were captured or just interacted with the surface of the endothelial cells (Fig. 8). This result apart of showing that hepatic EVs could be incorporated by the endothelium of pulmonary artery, also support that EV-mediated arginine depletion occur inside the endothelium, where eNOS is localized.

## Discussion

Although several works have already shown the presence of enzymatic activities associated to EVs including acetylcholinesterase^19^, carboxylesterase^11^, ATP- and 5′ AMP-phosphohydrolytic activity^20^, no much information of the integrated actions of the enzymatic content of EVs has been reported so far. In the current work, by applying an untargeted approach we focused on the global metabolic effect of the hepatocyte-released EVs on the composition of the serum, one of the two bodily fluids (the second is the bile) into which the hepatocytes can release their vesicles. Our method for the isolation of the EVs, including filtration through 220-nm pore filters and two consecutive ultracentrifugation steps, favored the enrichment in small EVs (below 220 nm). Our group has already performed a deep molecular characterization of these vesicles^1^,^11^,^21^. We have shown that, on the basis of their protein content, size range, and density, our vesicles population mostly consists of exosomes and small microvesicles, according to the ISEV standards^22^. In the current work, we examined the function of these vesicles. Given their complex enzyme cargo^1^,^11^, we decided to employ a comprehensive and unbiased metabolomics approach to analyze their global effects on the metabolite composition of the serum. We focused on the metabolites significantly and differentially expressed in the serum samples incubated at 37 °C with and without EVs. In concordance with the enzymatic diversity of the small hepatic EVs^1^,^11^, we identified changes in the abundance of metabolites of different chemical nature. We detected changes in the levels of a purine-related compound (deoxyinosine), a vitamin (ascorbic acid), 2 lipids (glycerophosphocholine and lysophosphatidylcholine), and several amino acids and related compounds (arginine, N-methyl-L-arginine, ornithine, citrulline, glutamate, 3-methyl-L-histidine, glutamyl-alanine, and L-anserine).

The most dramatic change was observed in the serum levels of the deoxyinosine. The abundance of this purine nucleoside increased by an average of 4-fold after the incubation with hepatic EVs. Interestingly, purine nucleosides can be used by mammalian cells as an energy source to replenish ATP; there is growing evidence indicating that they exert a protective effect during stress conditions^23^,^24^,^25^,^26^. The proposed mechanism involves the pentose phosphate pathway and glycolysis; the sugar moieties of the nucleosides are converted to the molecules that can be used as an energy source. It has been shown recently that the hepatoma cell line Huh-7 can use deoxyinosine as an energy source under glucose deprivation conditions and in the presence of FCCP (an inhibitor of mitochondrial respiration)^26^. Our discovery of hepatic EVs generating deoxyinosine suggests that one of the possible roles of these vesicles is generate the energy substrates in the extracellular environment during the response to injury. However, further investigation is required to confirm the fuel potential of hepatic EVs suggested by our study.

Another two metabolites whose serum levels increased due to the enzymatic activity of hepatic EVs were methyl-histidine and anserine. These two metabolites are involved in the same metabolic pathway. Methyl-histidine is the precursor of anserine, a dipeptide that acts as a natural antioxidant against reactive oxygen species^27^. We also found increased levels of 5-L-glutamyl-L-alanine, a substrate of the enzyme gamma-glutamyl transpeptidase. This enzyme plays a key role in the synthesis and metabolism of extracellular glutathione, another major natural antioxidant^28^. Moreover, we detected a decrease in the serum levels of ascorbic acid (vitamin C), also a natural antioxidant^29^.

Our results are in accord with reports showing the pro-regenerative role of liver-derived EVs^3^,^4^,^5^,^6^. It is likely that, in the extracellular environment, the hepatic EVs can provide energy substrates and are involved in oxidative stress response to achieve tissue regeneration.

Interestingly, we found that the levels of four metabolites associated with the nitric oxide metabolism, arginine, ornithine, citrulline, and N-methyl-L-arginine (an inhibitor of nitric oxide synthase), were affected by the incubation with hepatic EVs. The presence of Arg1 in EVs^1^ explains the consumption of the serum arginine. Using immunolabeling electron microscopy, we observed the presence of Arg1 associated to the membrane, a phenomenon that has been reported in red blood cells, mediated by the protein Flotillin-1^30^, a protein present as well in hepatocytes-derived EVs^21^. An increase in the serum arginase activity caused by hepatotoxicity is a well-known phenomenon^31^,^32^. It has also been observed an increase of circulating EVs related to liver damage^33^. Besides, in the current work we observed also an increase of Arg1 activity in the EVs coming from injured hepatocytes. It has been proposed that the arginine depletion created by this enzyme is responsible for liver damage caused by ischemia and reperfusion^34^. Our study confirms that the hepatic EVs carrying active Arg1 might be one of the factors responsible for this local damage. Furthermore, acute and chronic liver diseases are often associated with complications in remote organs^35^. The lung is one of the organs that might be affected by hepatic pathogenic processes^36^. An acute lung injury is relatively common (20–30%) in patients with acute liver failure^37^. Cirrhosis and portal hypertension might be associated with pulmonary hypertension or hepatopulmonary syndrome^36^. Although several different mechanisms might be involved in such pulmonary complications, EVs are likely candidates for mediators of these remote organ complications of liver diseases. Indeed, the levels of circulating EVs increase in patients with cirrhosis^38^, pulmonary hypertension^39^,^40^, or acute lung injury^41^. Even though the cellular origin of these EVs is unclear, our data suggest that the hepatic EVs might induce pulmonary endothelial dysfunction by delivering their arginase cargo, and thereby propagating the injury to the lung.

Endothelial dysfunction is a common feature in pulmonary vascular diseases, including pulmonary hypertension^42^. To find out whether the EV-derived arginase has a role in the pulmonary vascular function, we analyzed the arterial reactivity to ACh in isolated PA incubated with EVs. The relaxant response to ACh was abolished by the eNOS inhibitor L-NAME, indicating its full dependence on NO. After treatment with EVs, the relaxant response to ACh was inhibited, suggesting a decreased bioavailability of NO. Notably, the arginase inhibitor NOR-NOHA prevented the endothelial dysfunction induced by EVs. This is consistent with a reduced NO synthesis caused by a diminished substrate (i.e., arginine) availability. However, co-incubation with NOR-NOHA had no effect on endothelium-dependent vasodilation in control PA. This result indicated a low basal vascular arginase activity under control conditions. The finding that NOR-NOHA improves endothelial dysfunction in patients with coronary artery disease, but does not affect endothelium-dependent vasodilation in control subjects^43^, supports this interpretation. Interestingly, the reduction in NO synthesis might be heightened *in vivo*; the serum contains N-methyl-L-arginine, which competes with arginine for the nitric oxide synthase. In fact, eNOS activity is strongly dependent on the arginine/N-methyl-L-arginine ratio^44^. In contrast, the responses to the NO donor, nitroprusside, were unaffected by EVs, ruling out their effect on the sensitivity of the smooth muscle cells to the NO/cGMP pathway activity. In these functional experiments, the analysis of vascular reactivity was carried out in the absence of EVs, and importantly under our experimental conditions some EVs were inside the endothelium of pulmonary artery. Thus, the results suggested that the arginase activity was closely attached to or integrated into the tissues during the pre-incubation with EVs. In addition, it is worthy to highlight that the incubation of the arteries with EVs had no effect in the contractile capabilities induced by acetylcholine following the inhibition of eNOS (by using L-NAME, Fig. 7E). This result allows discarding the degradation of acetylcholine as a possible mechanism of the EVs-mediated effect over the endothelium.

The main finding of our study is that the hepatic EVs are carriers of arginase, which can affect pulmonary vascular function. In agreement with this conclusion, the hepatotoxicant monocrotaline, in a broadly used model of pulmonary hypertension, increases the levels of both circulating EVs^45^ and the arginase concentration in the blood^46^. Interestingly, an increased arginase activity is also observed in the endothelium of PA from monocrotaline-treated rats^47^. Increased arginase expression/activity has also been found in other experimental models of pulmonary hypertension, including pulmonary hypertension induced by intermittent or chronic hypoxia^48^,^49^, bleomycin^50^, and hemolysis^51^, as well as in patients with pulmonary hypertension^52^,^53^. Our data are in agreement with the results of these studies, strongly suggesting that an increased breakdown of arginine by arginase contributes to nitric oxide deficiency and plays a prominent role in the pathophysiology of pulmonary hypertension.

## Conclusions

**Figure 9:**
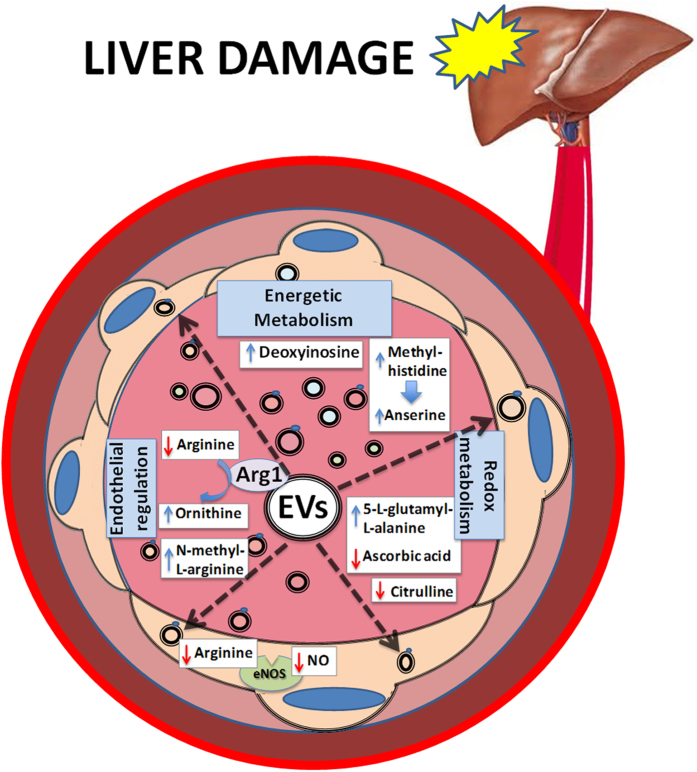
Schematic view of the global metabolic effects caused by hepatocyte-released EVs. Hepatic EVs can produce fuel molecules (e.g., deoxyinosine), alter oxidative environment (e.g., by changing the levels of anserine, 5-L-glutamyl-L-alanine, and ascorbic acid). They also reduce the arginine pool and increase the levels of eNOS inhibitor (N-methyl-L-arginine), affecting the endothelial function. Our findings indicate that the hepatic EVs transmit, coordinate, and integrate different types of metabolic activity (e.g., the energy, redox, and endothelial functions) in the extracellular environment. These properties of the EVs might be important for pathophysiological responses in the local areas and/or distant organs.

Hepatocytes secrete small EVs into the extracellular environment that are metabolically active and modify the levels of blood metabolites associated with the energy and redox metabolisms, and endothelial regulation (Fig. 9). They are involved in an arginase-dependent mechanism regulating the endothelial function locally and, possibly, at distant locations.

## Materials and Methods

### Animal procedures

All animal experimentation was conducted in accordance with Spanish guidelines for the care and use of laboratory animals, and protocols approved by the CIC bioGUNE Institute and the regional Basque Country ethical committee (ref. P-CBG-CBBA-3610). The surgery was performed under anesthetic gas Isoflurane (IsoFLO, Abbott Laboratories), and all efforts were made to minimize the suffering of the animals. Rat hepatocytes were obtained by liver perfusion of seven male 9-week-old Wistar rats. Briefly, after anesthetized the rat with isoflurane according to our institutions approved animal protocol, a laparotomy was performed to expose the liver and portal vein. The portal vein was cannulated for continuous perfusion with 150 ml Leffert’s buffer (Leffert’s buffer: HEPES 10 mM.KCl 3 mM, NaCl 130 mM, NaH2PO4-H2O mM,glucose 10 mM) with 1 mM EGTA. The infrahepatic inferior vena cava was open to allow the flow through. Then 150 ml of Leffert buffer was perfused and finally 150 ml of Leffert’s buffer containing 30,000 units of collagenase Type I (Worthington Biochemical Corp, Lakewood, NJ). After removing the catheters from the portal vein, the liver was explanted into a Petri dish, gently minced with scrapers and filtered through a cell strainer into a 50-mL tube. Hepatocytes were centrifuged at low speed (10 min at 500 × g) and plated as described below. In addition, from each animal, 1 ml of blood was collected. The serum was extracted using gel serum separator tubes (BD Microtainer SST Tubes), and stored at −80 °C until required. For the treatment with hepatotoxicants, 12 male Wistar rats (14 weeks old, body weight 300–400 g), were maintained in an environmentally controlled room at 22 °C on a 12-h light/dark cycle and provided with standard diet (Rodent Maintenance Diet, Harlan Teklad Global Diets 2014) and water *ad libitum*. The rats were randomly allocated to four groups. For the galactosamine (GalN) test, the test group (n = 3) received an intraperitoneal injection of 0.4 g/kg/5 ml of D(+)-galactosamine (2-amino-2-deoxy-D-galactose) hydrochloride (Sigma-Aldrich Chemical Co.). The animals in the control group (n = 3) were injected with the same volume of saline solution (5 ml/kg of 0.9% NaCl sterile). For acetaminophen challenge, the test group consisted of 3 animals, given acetaminophen (Sigma-Aldrich) orally at a dose of 1.2 g/kg/10 ml. The control group received the same volume of a mix of 0.25% Xanthan gum in 0.9% NaCl. In all groups, blood samples were collected during the sacrifice, 18 h after the treatment.

### Production and purification of small EVs

To obtain EVs, isolated primary rat hepatocytes were seeded on collagen-coated 150-mm dishes, at 15–30 million cells per dish. Primary hepatocytes were cultured in complete Dulbecco’s modified Eagle medium medium (DMEM), supplemented with 10% (v/v) of fetal bovine serum (FBS), 0.1 mg/mL streptomycin, and 100 units/mL penicillin (GIBCO Life Technologies Inc.), for 4 h at 37 °C and 5% CO_2_. The cells were washed twice with Dulbecco’s modified phosphate-buffered saline (PBS). Then, they were incubated for 36 h in 25 mM HEPES/complete DMEM medium, previously depleted of contaminating vesicles by overnight centrifugation at 110,000 × g^54^. Hepatotoxic drugs were added to the vesicle-depleted media. The concentrations employed were 10 mM galactosamine (GalN, Sigma-Aldrich), 10 mM acetaminophen (APAP, Sigma-Aldrich), or 400 μM diclofenac (DICLOF, Sigma-Aldrich). Afterward, the medium was collected, and EVs were isolated as previously described^55^. Briefly, the culture supernatant was centrifuged at 1,500 × g for 10 min to remove the lifted cells and cellular debris. The resultant supernatant was subjected to filtration on 0.22-μm pore filters, followed by ultracentrifugation at 10,000 × g and 100,000 × g for 30 min and 75 min, respectively. The pellet was resuspended in PBS, and again ultra-centrifuged at 100,000 × g for 75 min. The final pellet of small EVs was resuspended in 150 μL of PBS, and stored at −80 °C. For EV characterization, a further purification step was performed, using a sucrose density gradient separation^54^. Briefly, a continuous sucrose gradient ranging from 2.0 to 0.25 M sucrose in HEPES was prepared, and a 50-μg aliquot of isolated EVs was loaded on top. The samples were centrifuged in an SW40 rotor for 16 h at 100,000 × g. Then, 1-mL fractions were collected from top to bottom, using an Auto Densi-Flow Density Gradient Fractionator (Labconco, Kansas City, MO, USA). Each fraction was diluted in 2 mL of 20 mM HEPES (pH 7.4), placed in a polycarbonate thick-wall tube (Cat No 362305, Beckman Counter) and centrifuged for 1 h at 100,000 × g, at 4 °C, in a TLA-110 rotor. The pellets were resuspended in 25 μL of PBS and split into two portions, for the measurement of the arginase activity and identification of proteins using Western blotting. To purify EVs from the serum, 1 mL aliquots of serum were diluted with 2 mL of PBS and centrifuged at 10,000 × g for 30 min. The supernatant was further diluted and centrifuged at 100,000 × g for 2 h, and the pellet was resuspended in PBS and again ultra-centrifuged. The final pellet was resuspended in 50 μL of PBS. Bradford assay, with BSA as a standard, was used for protein quantitation.

### EV characterization by electron microscopy and nanoparticle-tracking analysis

For cryo-electron microscopy, EV preparations were directly adsorbed onto glow-discharged holey carbon grids (QUANTIFOIL, Germany). Grids were blotted at 95% humidity and rapidly plunged into liquid ethane with the aid of VITROBOT (Maastricht Instruments BV, The Netherlands). Vitrified samples were imaged at liquid nitrogen temperature using a JEM-2200FS/CR transmission electron microscope (JEOL, Japan) equipped with a field emission gun and operated at an acceleration voltage of 200 kV. For immunoelectron microscopy, EV preparations were fixed in 2% paraformaldehyde in PBS and deposited onto Formvar/carbon-coated EM grids. The vesicle-coated grids were washed twice with PBS (3 min each), twice with PBS/50 mM glycine, and finally blocked with PBS/0.5% BSA (15 min). Blocked grids were transferred to a drop of PBS/1% BSA containing antibody against Arg1 protein and incubated for 2 h. The grids were then washed with PBS/1% BSA five times for 3 min, incubated with 10-nm gold-labelled anti-rabbit secondary antibody in PBS/1% BSA at 4 °C overnight. Afterward, the grids were washed five times (for 3 min) in 100-μL drops of PBS, stained with 2% uranyl acetate and then viewed under a JEM-2200FS/CR transmission electron microscope.

Size distribution within EV preparations was analyzed using the nanoparticle-tracking analysis (NTA), by measuring the rate of Brownian motion in a NanoSight LM10 system (Malvern, U.K.). The system was equipped with a fast video-capture and particle-tracking software. NTA post-acquisition settings were the same for all samples. Each video was analyzed to give the mean, mode, and median vesicle size, as well as an estimate of the concentration.

### EV incubation and serum metabolite extraction

A schematic view of the serum and EV incubations and further metabolomics analysis is provided in Fig. 2. The sera of different animals were pooled, and aliquots of the same pool were used for all the incubations. Briefly, an Eppendorf tube containing 50 μL of rat serum was incubated with 5 μg (in 20 μL of PBS) of small EVs at 37 °C for 1 h, in a water bath. In parallel, another aliquot of 50 μL of the same rat serum and a 5-μg aliquot (in 20 μL of PBS) of small EVs were incubated separately at 37 °C for 1 h, cooled down on ice, and pooled immediately. After incubation, 60 μL of each sample was mixed with an equal volume of a mixture of methanol, water, and formic acid (75/24.9/0.1; v/v/v %). Subsequently, 120 μL of chloroform was added. The resulting suspensions were manually vortexed (twice for 2 seconds) and sonicated for 10 min at 4 °C. The solution was further agitated for 30 min at 1,400 rpm and 4 °C in a thermomixer. Next, the suspensions were centrifuged for 15 min at 13,000 rpm and 4 °C. The aqueous and organic phases were separated, and aliquots of 100 μL of both phases were evaporated to dryness in a speed-vacuum centrifuge. The resulting pellets were solubilized in 100 μL of a mixture of acetonitrile and water (60/40 v/v %). From these suspensions, 60-μL aliquots were used for the analysis. Aliquots of 40 μL of each extraction phase were pooled for quality control (QC) samples. The samples from the aqueous phase were analyzed first, followed by the samples from the organic phase. Seven independent replicates of each condition were performed.

### Chromatography and mass spectrometry (UPLC-MS)

During UPLC-MS analysis, all samples were stored in an ACQUITY Sample Manager (Waters Inc., Manchester, UK), at 4 °C. For the analysis of aqueous and organic phases, 2 different chromatographic methods were used, employing an ACQUITY UPLC system (Waters Inc.). The aqueous extractions were separated on a 100 × 2.1 mm, 1.7 μm, ACQUITY UPLC BEH Amide column (Waters Inc.) kept at 40 °C. The aqueous mobile phase (A1) consisted of 10 mM ammonium formate in water and formic acid (99.5/0.5 v/v%), and the organic mobile phase (B1) consisted of acetonitrile, water, and formic acid (99/0.5/0.5 v/v/v%). The chromatographic gradient used was as follows: from 20% A1 to 50% A1 for 6 min with a curved gradient (#9, as defined by the Waters MassLynx software), from 50% A1 to 99% A1 for 2 min in a linear gradient (#6), constant at 99% A1 for 1 min, and back to 20% A1 for 0.1 min (linear). Subsequently, the column was equilibrated for 2 min in 20% A1. The flow rate was 0.25 mL/min and the injection volume was 2 μL.

The organic extracts were separated on a 100 × 1.0 mm, 1.7 μm, ACQUITY UPLC BEH C8 column (Waters Inc.) at 40 °C. The aqueous mobile phase (A2) consisted of water and formic acid (99.9/0.1 v/v %), and the organic mobile phase (B2) consisted of acetonitrile, water, and formic acid (99.8/0.1/0.1 v/v/v %). The chromatographic gradient used was as follows: from 50% A2 to 1% A2 for 7 min in a linear gradient, constant at 1% A2 for 1.4 min, and back at 50% A1 for 0.2 min. Subsequently, the column was equilibrated for 3 min in 50% A1. The flow rate was 0.10 mL/min and the injection volume was 2 μL.

The mass spectrometer settings were identical for both extraction phases. All samples were analyzed on a SYNAPT G2 HDMS Time-of-Flight instrument (Waters Inc.) The instrument was operated first in the ESI + (electrospray ionization) and next in ESI- mode with capillary voltages of 500 V. The extraction cone voltage was 5 V and sampling cone voltage was 25 V. The source temperature was 120 °C and the capillary temperature was 450 °C. Two scan functions were used: one for low collision energy (4 V) and one for high collision energy (25 V). Samples were examined in full scan mode from 50 to 1200 Da. Drift in m/z values was corrected for by the lock mass of a Leu-enkephalin signal at m/z 556.2771. Both polarities were tuned on the Leu-enkephalin (intensity 1e5) to an FWHM of 21,000. All data were processed using the MarkerLynx application manager for MassLynx 4.1 software (Waters Corp., Milford, USA). The LC/MS data were peak-detected and noise-reduced. A list of intensities (chromatographic peak areas) of the peaks detected was then generated. To identify the signals that differed between the sera incubated with and without EVs, multiple univariate analysis was performed. We used the paired *t*-test or Wilcoxon test depending on the normality of the distribution of each metabolite among the samples. Afterward, the peaks with significant differences between the two groups were manually re-integrated, and the values were normalized and QC-corrected. A plausible identification based on the calculated mass, isotopic composition, and fragmentation data was achieved by a search against the metabolite databases Human Metabolome Database (HMDB), METLIN, and Madison Metabolomics Consortium Database. The positively identified metabolites were further confirmed using the commercial chemical standards. The relevance of those metabolites in different metabolic pathways was evaluated using KEGG Color Pathway tool (http://www.genome.jp/kegg/tool/map_pathway2.html).

### Western blot analysis

After the density gradient, 30 μL of PBS-resuspended EVs were mixed with 10 μL of 4 × NuPAGE LDS Sample Buffer (Thermo Fisher Scientific). The samples were incubated for 5 min at 37 °C, 65 °C, and 95 °C, and separated on 4–12% pre-casted gels (from Thermo Scientific, Inc.). For kidney extraction, 50-mg portions of rat tissue were disrupted in a Precellys homogenizer, in the cell lysis buffer (50 mM Tris-HCl pH 7.5, 300 mM NaCl, 0.1% Triton X-100) containing a cocktail of protease inhibitors (Sigma-Aldrich). The homogenate was centrifuged, the supernatant collected, and the protein quantified. 10-μg of the protein extract was used in the Western blot analysis. The samples were transferred to PVDF membranes and blocked for 1 h (5% milk and 0.05% Tween-20 in PBS), the primary antibody was added overnight, followed by PBS wash and the application of secondary HRP-conjugated antibody. All proteins were detected under non-reducing conditions. Chemiluminescent bands were detected with Clarity™ ECL Western Blotting Substrate (Bio-rad Laboratories, Inc). Mouse monoclonal antibodies were purchased from the following vendors: Mouse monoclonal antibodies against Aip1/Alix, Rab8 (clone 4/Rab8), and CD63 (clone AD1) were purchased from BD Biosciences; rabbit polyclonal antibody against CD26 was purchased from Abcam. Armenian hamster anti-mouse CD81 (clone Eat2) was purchased from Bio-Rad and rabbit polyclonal antibodies against Arg1 and Arg2, from GeneTex. Mouse monoclonal antibody against Hsp70 (clone BRM22) was obtained from Santa Cruz Biotechnology, Inc.

### Arginase and GOT (glutamic-oxaloacetic transaminase) activities

To measure the arginase activity, we employed the Arginase Assay Kit (Sigma-Aldrich Chemical Co.) following the manufacturer’s instructions. Before the assay, urea was removed from the samples using a 10-kDa Microcon Molecular Weight Cut-Off Filter (Millipore). The arginase activity values were calculated according to the manufacturer’s instructions and referenced to the values of their own urea standard to calculate the concentration. GOT activity in the serum samples was measured using the Infinity™ ALT(GPT) Liquid Stable Reagent (Thermo Scientific), following the manufacturer’s instructions.

### Dissection of the rat pulmonary artery and recording the vascular reactivity

Secondary branches of pulmonary arteries (PA; internal diameter <0.8 mm) were dissected free of the surrounding tissue and cut into rings (1.7–2-mm long). The PA rings were incubated for 2 h in 0% FBS DMEM in the absence (control) or presence of EV suspensions (50 μg/mL). In some experiments, rings were co-treated with the arginase inhibitor N^ω^-hydroxy-nor-L-Arginine (NOR-NOHA, 10 ng/mL). EV preparations were obtained from six different rats and incubated with PA rings collected from at least three independent rats. PA rings were mounted in a wire myograph with Krebs solution bubbled with 95% O_2_–5% CO_2_, as previously reported^56^. After equilibration, arterial rings were first stimulated with KCl (80 mM). Then, the endothelial function was assessed in PA rings pre-contracted with phenylephrine (10^−6^ M), and a cumulative dose-response curve to acetylcholine (ACh, 10^−9^ to 10^−5^ M) was obtained. Afterward, the vascular rings were washed during a 30-min recovery period and treated with the endothelial nitric oxide (eNOS) inhibitor L-NAME (L-NG-nitroarginine methyl ester; 10^−4^ M) for 20 min before exposing them again to phenylephrine (10^−6^ M). The second dose-response curve to ACh was obtained. Finally, the relaxant effects of sodium nitroprusside (SNP; 10^−9^ to 10^−5^ M) were assessed.

### Labeling of EVs with liphophilic fluorophore DiI

EVs were labeled by using the liphophilic fluorescence dye, DiI (Molecular Probes) following the manufacture’s instructions. Briefly, EVs were incubated with the dye at room temperature. After 30 minutes of incubation samples were ultracentrifuged at 100,000 × g for 75 min. Pellets were washed with PBS and again ultracentrifuged at 100,000 × g for 75 min. Labelled-EVs were resuspended in PBS. In parallel same volume of the dye was incubated in the absence of EVs and subjected to same incubation periods, ultracentrifugation and wash steps previously described. This latter sample was used to control the residual non-vesicular incorporated DiI label that could be co-purified during the ultracentrifugation process.

### Analysis of the incorporation of hepatic EVs into endothelium of pulmonary artery

Arteries were dissected and incubated with DiI-labeled EVs or residual-DiI control for 2-hours, then fixed with 2%-Formaldheyde, PBS-washed and included in OCT for histological analysis. Sections of 10 microns were mounted on DAPI-containing Fluoromount G (Southern Biotech) and analyzed in a Leica Confocal Microscopy. Elastin autofluorescence was triggered by 488 nm laser line. All images were taken under same conditions and magnification.

### Statistics

Principal component analysis (PCA) of metabolomics data was performed using R-software. The first three principal components were plotted, representing the most important metabolic variation in the samples captured by the analysis. Univariate analysis was performed by applying paired *t*-test or Wilcoxon test depending on the normality of the distribution of each metabolite among the samples. Arginase activities in the two groups, with or without arginase inhibitor, were compared using the *t*-test (Fig. 4). ANOVA was performed to compare the groups shown in Figs 6 and 7.

## Additional Information

**How to cite this article:** Royo, F. *et al*. Hepatocyte-secreted extracellular vesicles modify blood metabolome and endothelial function by an arginase-dependent mechanism. *Sci. Rep.* **7**, 42798; doi: 10.1038/srep42798 (2017).

**Publisher's note:** Springer Nature remains neutral with regard to jurisdictional claims in published maps and institutional affiliations.

## Supplementary Material

Supplementary Information

## Figures and Tables

**Figure 1:**
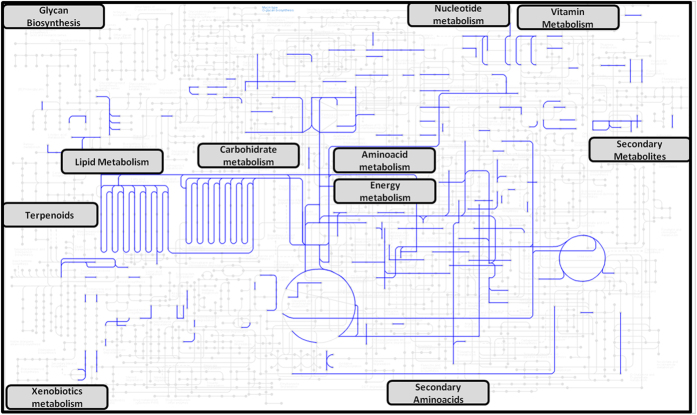
Wide representation of metabolic pathways in hepatocyte-secreted EVs. Enzymes previously described in hepatocyte-secreted EVs^11^ were mapped in the complete map of cellular metabolic pathways by using KEGG Mapper-search & color pathway tool. Enzymes detected in hepatic EVs are highlighted in blue. Cellular pathways that are represented in hepatocyte-secreted EVs are indicated with labels.

**Table 1:** Serum metabolites modified by small EVs secreted by hepatocytes.

Metabolite	%Deviation^a^ (±95% CIs)	*p*-value
Ascorbic acid	−25% (±23%)	0.04
Glutamate	4% (±4%)	0.04
Methyl-histidine	18% (±6%)	0.0003
Anserine	34% (±14%)	0.001
Ornithine	69% (±17%)	0.00005
Arginine	−45% (±19%)	0.001
Methyl-arginine	10% (±7%)	0.013
Glutamyl-alanine	6% (±4%)	0.006
Citrulline	−9% (±8%)	0.04
Deoxyinosine	295% (±163%)	0.004
Glycerophosphocholine	8% (±5%)	0.004
LPC(14:0)	−15% (±13%)	0.032

^a^% Deviation = 100% × (A_inc_ − A_ctr_)/A_ctr_, where A_inc_ is the peak area for the serum incubated with EVs and A_ctr_, the peak area for the control incubations (*i.e*., serum and EVs incubated separately, which could be considered as the initial level of the specific metabolite).

**Table 2:** Metabolic pathways represented by the metabolites altered by the EVs.

Pathway name^a^	Total^b^	Hits^c^
Arginine and proline metabolism	44	3
Histidine metabolism	15	2
Glutathione metabolism	26	2
Glycerophospholipid metabolism	30	2
Ascorbate and aldarate metabolism	9	1
Nitrogen metabolism	9	1
Aminoacyl-tRNA biosynthesis	67	2
beta-Alanine metabolism	19	1
Purine metabolism	68	1

^a^Pathways were analyzed using KEGG color and pathway tool.

^b^Number of metabolites involved in the pathway.

^c^Number of metabolites detected in the study.

## References

[b1] Conde-Vancells J. . Characterization and comprehensive proteome profiling of exosomes secreted by hepatocytes. Journal of proteome research 7, 5157–5166 (2008). 19367702 10.1021/pr8004887 PMC2696236

[b2] Gonzalez E. & Falcon-Perez J. M. Cell-derived extracellular vesicles as a platform to identify low-invasive disease biomarkers. Expert review of molecular diagnostics 15, 907–923, doi: 10.1586/14737159.2015.1043272 (2015). 25948243

[b3] Herrera M. B. . Human liver stem cells improve liver injury in a model of fulminant liver failure. Hepatology 57, 311–319, doi: 10.1002/hep.25986 (2013). 22829291

[b4] Herrera M. B. . Human liver stem cell-derived microvesicles accelerate hepatic regeneration in hepatectomized rats. Journal of cellular and molecular medicine 14, 1605–1618, doi: 10.1111/j.1582-4934.2009.00860.x (2010). 19650833 PMC3060338

[b5] Herrera Sanchez M. B. . Human liver stem cells and derived extracellular vesicles improve recovery in a murine model of acute kidney injury. Stem cell research & therapy 5, 124, doi: 10.1186/scrt514 (2014). 25384729 PMC4446072

[b6] Nojima H. . Hepatocyte exosomes mediate liver repair and regeneration via sphingosine-1-phosphate. Journal of hepatology, doi: 10.1016/j.jhep.2015.07.030 (2015). PMC4843792 26254847

[b7] Hirsova P. . Lipid-Induced Signaling Causes Release of Inflammatory Extracellular Vesicles From Hepatocytes. Gastroenterology 150, 956–967, doi: 10.1053/j.gastro.2015.12.037 (2016). 26764184 PMC4808464

[b8] Conde-Vancells J., Gonzalez E., Lu S. C., Mato J. M. & Falcon-Perez J. M. Overview of extracellular microvesicles in drug metabolism. Expert opinion on drug metabolism & toxicology 6, 543–554, doi: 10.1517/17425251003614766 (2010). 20192903 PMC2856761

[b9] Holman N. S., Mosedale M., Wolf K. K., LeCluyse E. L. & Watkins P. B. Sub-toxic alterations in hepatocyte-derived exosomes: an early step in drug-induced liver injury? Toxicological sciences: an official journal of the Society of Toxicology, doi: 10.1093/toxsci/kfw047 (2016). PMC4880137 26962055

[b10] Yanez-Mo M. . Biological properties of extracellular vesicles and their physiological functions. Journal of extracellular vesicles 4, 27066, doi: 10.3402/jev.v4.27066 (2015). 25979354 PMC4433489

[b11] Rodriguez-Suarez E. . Quantitative proteomic analysis of hepatocyte-secreted extracellular vesicles reveals candidate markers for liver toxicity. Journal of proteomics 103, 227–240, doi: 10.1016/j.jprot.2014.04.008 (2014). 24747303 PMC5119459

[b12] Gonzalez E. . Serum UPLC-MS/MS metabolic profiling in an experimental model for acute-liver injury reveals potential biomarkers for hepatotoxicity. Metabolomics: Official journal of the Metabolomic Society 8, 997–1011, doi: 10.1007/s11306-011-0329-9 (2012). 23139648 PMC3490499

[b13] Weckwerth W. Metabolomics: an integral technique in systems biology. Bioanalysis 2, 829–836, doi: 10.4155/bio.09.192 (2010). 21083277

[b14] Royo F. . Metabolically active extracellular vesicles released from hepatocytes under drug-induced liver-damaging conditions modify serum metabolome and might affect different pathophysiological processes. European journal of pharmaceutical sciences: official journal of the European Federation for Pharmaceutical Sciences, doi: 10.1016/j.ejps.2016.10.020 (2016). 27771515

[b15] Caldwell R. B., Toque H. A., Narayanan S. P. & Caldwell R. W. Arginase: an old enzyme with new tricks. Trends Pharmacol Sci 36, 395–405, doi: 10.1016/j.tips.2015.03.006 (2015). 25930708 PMC4461463

[b16] Durante W., Johnson F. K. & Johnson R. A. Arginase: a critical regulator of nitric oxide synthesis and vascular function. Clinical and experimental pharmacology & physiology 34, 906–911, doi: 10.1111/j.1440-1681.2007.04638.x (2007). 17645639 PMC1955221

[b17] Boucher J. L., Moali C. & Tenu J. P. Nitric oxide biosynthesis, nitric oxide synthase inhibitors and arginase competition for L-arginine utilization. Cell Mol Life Sci 55, 1015–1028 (1999). 10484661 10.1007/s000180050352 PMC11147020

[b18] Pernow J., Kiss A., Tratsiakovich Y. & Climent B. Tissue-specific up-regulation of arginase I and II induced by p38 MAPK mediates endothelial dysfunction in type 1 diabetes mellitus. British journal of pharmacology 172, 4684–4698, doi: 10.1111/bph.13242 (2015). 26140333 PMC4594272

[b19] Johnstone R. M., Adam M., Hammond J. R., Orr L. & Turbide C. Vesicle formation during reticulocyte maturation. Association of plasma membrane activities with released vesicles (exosomes). The Journal of biological chemistry 262, 9412–9420 (1987). 3597417

[b20] Clayton A., Al-Taei S., Webber J., Mason M. D. & Tabi Z. Cancer exosomes express CD39 and CD73, which suppress T cells through adenosine production. Journal of immunology 187, 676–683, doi: 10.4049/jimmunol.1003884 (2011). 21677139

[b21] Royo F. . Transcriptome of extracellular vesicles released by hepatocytes. PloS one 8, e68693, doi: 10.1371/journal.pone.0068693 (2013). 23874726 PMC3708910

[b22] Lotvall J. . Minimal experimental requirements for definition of extracellular vesicles and their functions: a position statement from the International Society for Extracellular Vesicles. J Extracell Vesicles 3, 26913, doi: 10.3402/jev.v3.26913 (2014). 25536934 PMC4275645

[b23] Shin C. Y. . Adenosine and purine nucleosides protect rat primary astrocytes from peroxynitrite-potentiated, glucose deprivation-induced death: preservation of intracellular ATP level. Exp Neurol 176, 175–182 (2002). 12093094 10.1006/exnr.2002.7913

[b24] Szoleczky P. . Identification of agents that reduce renal hypoxia-reoxygenation injury using cell-based screening: purine nucleosides are alternative energy sources in LLC-PK1 cells during hypoxia. Arch Biochem Biophys 517, 53–70, doi: 10.1016/j.abb.2011.11.005 (2012). 22100704 PMC4676579

[b25] Balestri F. . Purine and pyrimidine nucleosides preserve human astrocytoma cell adenylate energy charge under ischemic conditions. Neurochem Int 50, 517–523, doi: 10.1016/j.neuint.2006.10.005 (2007). 17126452

[b26] Salleron L. . DERA is the human deoxyribose phosphate aldolase and is involved in stress response. Biochim Biophys Acta 1843, 2913–2925, doi: 10.1016/j.bbamcr.2014.09.007 (2014). 25229427

[b27] Yanai N., Shiotani S., Hagiwara S., Nabetani H. & Nakajima M. Antioxidant combination inhibits reactive oxygen species mediated damage. Biosci Biotechnol Biochem 72, 3100–3106, doi: 10.1271/bbb.80159 (2008). 19060409

[b28] Hanigan M. H. Gamma-glutamyl transpeptidase: redox regulation and drug resistance. Adv Cancer Res 122, 103–141, doi: 10.1016/B978-0-12-420117-0.00003-7 (2014). 24974180 PMC4388159

[b29] Bielli A., Scioli M. G., Mazzaglia D., Doldo E. & Orlandi A. Antioxidants and vascular health. Life Sci 143, 209–216, doi: 10.1016/j.lfs.2015.11.012 (2015). 26585821

[b30] Jiang M. . Arginase-flotillin interaction brings arginase to red blood cell membrane. FEBS letters 580, 6561–6564, doi: 10.1016/j.febslet.2006.11.003 (2006). 17113085

[b31] Bailey W. J. . A performance evaluation of three drug-induced liver injury biomarkers in the rat: alpha-glutathione S-transferase, arginase 1, and 4-hydroxyphenyl-pyruvate dioxygenase. Toxicological sciences: an official journal of the Society of Toxicology 130, 229–244, doi: 10.1093/toxsci/kfs243 (2012). 22872058

[b32] Ozcelik E., Uslu S., Burukoglu D. & Musmul A. Chitosan and blueberry treatment induces arginase activity and inhibits nitric oxide production during acetaminophen-induced hepatotoxicity. Pharmacognosy magazine 10, S217–224, doi: 10.4103/0973-1296.133234 (2014). 24991095 PMC4078330

[b33] Brodsky S. V. . Dynamics of circulating microparticles in liver transplant patients. Journal of gastrointestinal and liver diseases: JGLD 17, 261–268 (2008). 18836617

[b34] Jeyabalan G. . Arginase blockade protects against hepatic damage in warm ischemia-reperfusion. Nitric oxide: biology and chemistry/official journal of the Nitric Oxide Society 19, 29–35, doi: 10.1016/j.niox.2008.04.002 (2008). PMC2527546 18456004

[b35] Matuschak G. M. Liver-lung interactions in critical illness. New Horiz 2, 488–504 (1994). 7804798

[b36] Rodriguez-Roisin R., Krowka M. J., Herve P., Fallon M. B. & Committee, E. R. S. T. F. P.-H. V. D. S. Pulmonary-Hepatic vascular Disorders (PHD). Eur Respir J 24, 861–880, doi: 10.1183/09031936.04.00010904 (2004). 15516683

[b37] Baudouin S. V., Howdle P., O’Grady J. G. & Webster N. R. Acute lung injury in fulminant hepatic failure following paracetamol poisoning. Thorax 50, 399–402 (1995). 7785015 10.1136/thx.50.4.399 PMC474296

[b38] Rautou P. E. . Abnormal plasma microparticles impair vasoconstrictor responses in patients with cirrhosis. Gastroenterology 143, 166–176 e166, doi: 10.1053/j.gastro.2012.03.040 (2012). 22465620

[b39] Amabile N. . Cellular microparticles in the pathogenesis of pulmonary hypertension. Eur Respir J 42, 272–279, doi: 10.1183/09031936.00087212 (2013). 23258792

[b40] Tual-Chalot S. . Circulating microparticles from pulmonary hypertensive rats induce endothelial dysfunction. Am J Respir Crit Care Med 182, 261–268, doi: 10.1164/rccm.200909-1347OC (2010). 20339146

[b41] McVey M., Tabuchi A. & Kuebler W. M. Microparticles and acute lung injury. Am J Physiol Lung Cell Mol Physiol 303, L364–381, doi: 10.1152/ajplung.00354.2011 (2012). 22728467

[b42] Klinger J. R., Abman S. H. & Gladwin M. T. Nitric oxide deficiency and endothelial dysfunction in pulmonary arterial hypertension. Am J Respir Crit Care Med 188, 639–646, doi: 10.1164/rccm.201304-0686PP (2013). 23822809

[b43] Shemyakin A. . Arginase inhibition improves endothelial function in patients with coronary artery disease and type 2 diabetes mellitus. Circulation 126, 2943–2950, doi: 10.1161/CIRCULATIONAHA.112.140335 (2012). 23183942

[b44] Tsikas D., Boger R. H., Sandmann J., Bode-Boger S. M. & Frolich J. C. Endogenous nitric oxide synthase inhibitors are responsible for the L-arginine paradox. FEBS Lett 478, 1–3 (2000). 10922458 10.1016/s0014-5793(00)01686-0

[b45] Aliotta J. M. . Exosomes induce and reverse monocrotaline-induced pulmonary hypertension in mice. Cardiovasc Res 110, 319–330, doi: 10.1093/cvr/cvw054 (2016). 26980205 PMC4872877

[b46] Saitoh W., Yamauchi S., Watanabe K., Takasaki W. & Mori K. Metabolomic analysis of arginine metabolism in acute hepatic injury in rats. J Toxicol Sci 39, 41–50 (2014). 24418708 10.2131/jts.39.41

[b47] Sasaki A., Doi S., Mizutani S. & Azuma H. Roles of accumulated endogenous nitric oxide synthase inhibitors, enhanced arginase activity, and attenuated nitric oxide synthase activity in endothelial cells for pulmonary hypertension in rats. Am J Physiol Lung Cell Mol Physiol 292, L1480–1487, doi: 10.1152/ajplung.00360.2006 (2007). 17322279

[b48] Nara A. . Pulmonary arterial hypertension in rats due to age-related arginase activation in intermittent hypoxia. Am J Respir Cell Mol Biol 53, 184–192, doi: 10.1165/rcmb.2014-0163OC (2015). 25490411

[b49] Stenmark K. R., Tuder R. M. & El Kasmi K. C. Metabolic reprogramming and inflammation act in concert to control vascular remodeling in hypoxic pulmonary hypertension. J Appl Physiol (1985) 119, 1164–1172, doi: 10.1152/japplphysiol.00283.2015 (2015). 25930027 PMC4816410

[b50] Grasemann H. . Arginase inhibition prevents bleomycin-induced pulmonary hypertension, vascular remodeling, and collagen deposition in neonatal rat lungs. Am J Physiol Lung Cell Mol Physiol 308, L503–510, doi: 10.1152/ajplung.00328.2014 (2015). 25595650

[b51] Hsu L. L. . Hemolysis in sickle cell mice causes pulmonary hypertension due to global impairment in nitric oxide bioavailability. Blood 109, 3088–3098, doi: 10.1182/blood-2006-08-039438 (2007). 17158223 PMC1852224

[b52] Kao C. C. . Arginine metabolic endotypes in pulmonary arterial hypertension. Pulm Circ 5, 124–134, doi: 10.1086/679720 (2015). 25992277 PMC4405713

[b53] Morris C. R. . Dysregulated arginine metabolism, hemolysis-associated pulmonary hypertension, and mortality in sickle cell disease. JAMA 294, 81–90, doi: 10.1001/jama.294.1.81 (2005). 15998894 PMC2065861

[b54] Thery C., Amigorena S., Raposo G. & Clayton A. Isolation and characterization of exosomes from cell culture supernatants and biological fluids. Current protocols in cell biology / editorial board, Juan S. Bonifacino … [*et al*.] Chapter 3, Unit 3 22, doi: 10.1002/0471143030.cb0322s30 (2006). 18228490

[b55] Raposo G. . B lymphocytes secrete antigen-presenting vesicles. J Exp Med 183, 1161–1172 (1996). 8642258 10.1084/jem.183.3.1161 PMC2192324

[b56] Moral-Sanz J. . Different patterns of pulmonary vascular disease induced by type 1 diabetes and moderate hypoxia in rats. Experimental physiology 97, 676–686, doi: 10.1113/expphysiol.2011.062257 (2012). 22247283

