# Supplementary material for: Hepatocyte-secreted extracellular vesicles modify blood metabolome and endothelial function by an arginase-dependent mechanism
Source: Sci Rep. 2017 Feb 17;7:42798. doi: 10.1038/srep42798 (PMC5314384; doi:10.1038/srep42798)

Title:

Hepatocyte-secreted extracellular vesicles modify blood metabolome and endothelial function by an arginase-dependent mechanism

Authors:

Felix Royo[1], Laura Moreno[2], Justyna Mleczko[1], Laura Palomo[1], Esperanza Gonzalez[1], Diana Cabrera[3], Angel Cogolludo[2], Francisco Perez Vizcaino[2], Sebastiaan van-Liempd[3] and Juan M Falcon-Perez[1,3,4]

Affiliations:

[1] Exosomes Laboratory. CIC bioGUNE, CIBEREHD, Derio 48160 Bizkaia, Spain.

[2] Department of Pharmacology, School of Medicine, University Complutense of Madrid, 28040 Madrid, Spain. CIBERES. Instituto de Investigación Sanitaria Gregorio Marañón (IISGM)

[3] Metabolomics platform, CIC bioGUNE, CIBERehd, , Derio 48160 Bizkaia, Spain

[4] IKERBASKE Basque Foundation for science, Bilbao, 48013 Bizkaia, Spain

Supplemental Figure Legends

Supplemental Figure 1. Arginase and GOT activity in the serum of rats treated with vehicle (control) or indicated hepatotoxicants**. (A)** Arginase activity (U/L) was normalized to the activity detected in the control. The error bars represent S.E.M (n = 3) (*t*-test; *p*-values for APAP = 0.001 and GalN < 0.001). **(B)** Correlation between GOT transaminase and arginase activities in the serum of rats treated with vehicle (control) or indicated hepatotoxicants (n = 3). Correlation coefficients for linear regression are indicated.

Supplemental Figure 2. Endothelium-independent relaxation of pulmonary arteries (PA) is preserved following the treatment with EVs. Concentration-dependent relaxation induced by the endothelium-independent vasodilator, sodium nitroprusside (SNP), in control (n = 6) or EV-treated (50 µg/mL; n = 6) rat PA rings incubated in the absence **(A)** or presence **(B)** of NOR-NOHA (10 ng/mL; n = 6). Results are expressed as a percentage of the relaxation induced by SNP; they were analyzed using the repeated measures ANOVA.

Supplemental Figure 1

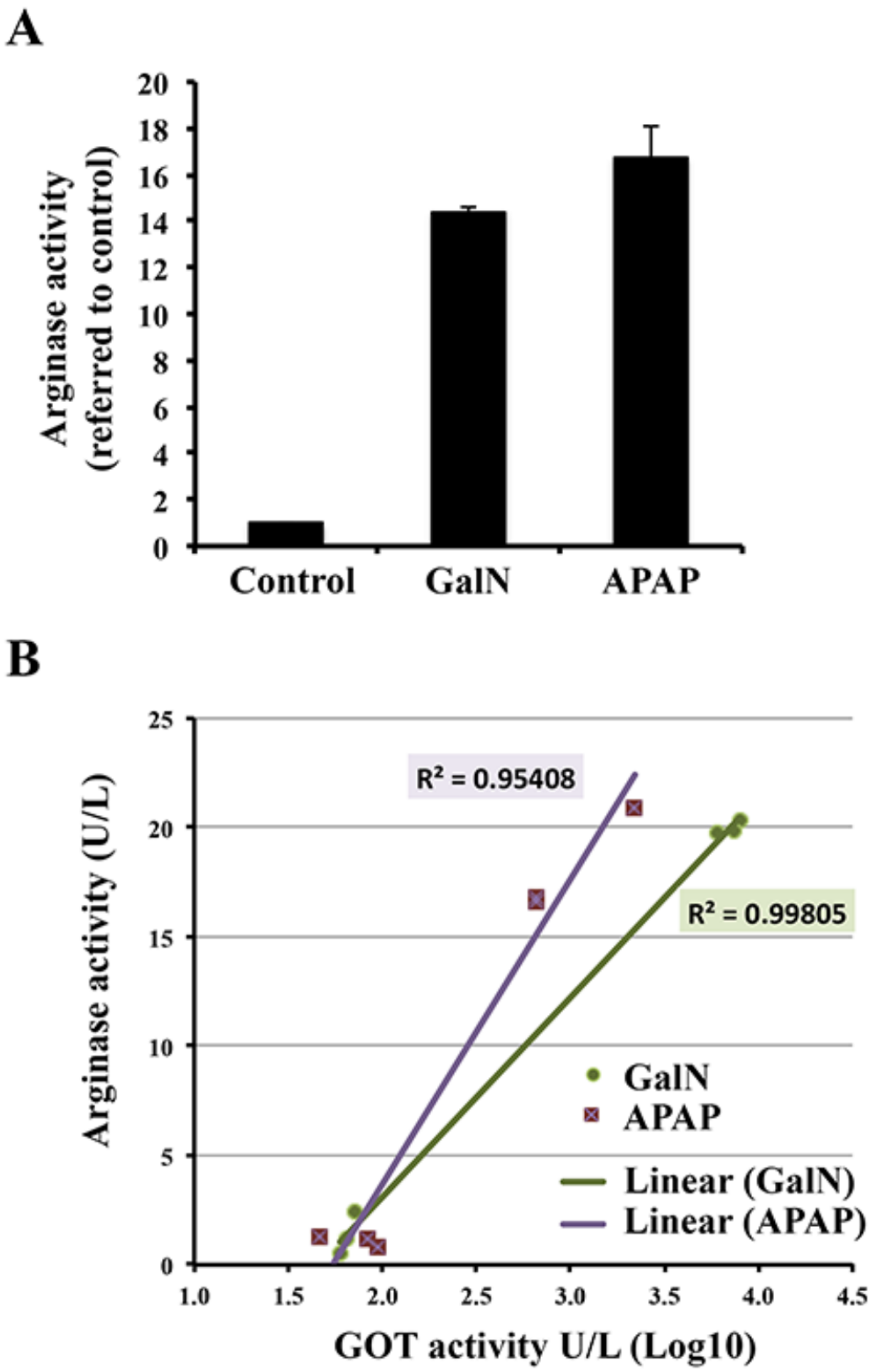

Supplemental Figure 2

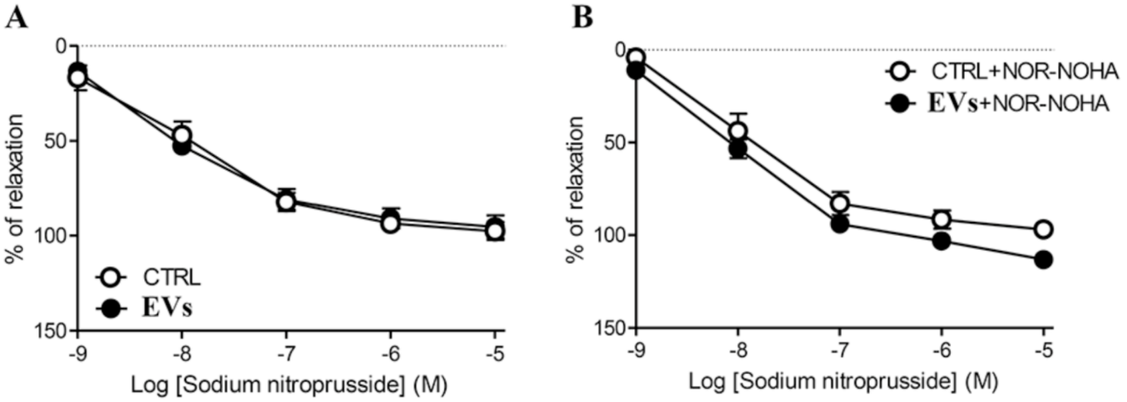

Supplement: Supplementary Information [file srep42798-s1.pdf]
